# The Influence of Initial Microstructure on the Microstructure and Mechanical Properties of Ductile Iron After Nanostructurisation Heat Treatment

**DOI:** 10.3390/nano15221710

**Published:** 2025-11-12

**Authors:** Emilia Skołek, Paweł Skoczylas, Kamil Wasiluk, Wiesław A. Świątnicki, Andrzej N. Wieczorek, Dawid Myszka

**Affiliations:** 1 Faculty of Materials Science and Engineering, Warsaw University of Technology, Wołoska 141, 02-507 Warsaw, Poland; kamil.wasiluk@nanostal.com (K.W.); wieslaw.swiatnicki@pw.edu.pl (W.A.Ś.); 2 Faculty of Mechanical and Industrial Engineering, Warsaw University of Technology, Narbutta 85, 02-524 Warsaw, Poland; pawel.skoczylas@pw.edu.pl (P.S.); dawid.myszka@pw.edu.pl (D.M.); 3 Faculty of Mining, Safety Engineering and Industrial Automation, Silesian University of Technology, Akademicka 2 Street, 44-100 Gliwice, Poland; andrzej.n.wieczorek@polsl.pl

**Keywords:** austempered ductile iron, austempering, nanoausferritic matrix, nanostructurisation, pre-heat treatment

## Abstract

In this study, the microstructure and mechanical properties of ductile cast iron subjected to a novel austempering treatment were investigated. The methodology was based on prolonged isothermal annealing within the low-temperature range of the bainitic transformation. The experiments were carried out on samples with two different initial microstructures: pearlitic–ferritic and ausferritic. Two long-term austempering treatment variants were designed and tested: single (AT-1) and double (AT-2). In both cases, the heat treatment led to the production of a nanoausferritic microstructure, characterized by exceptionally thin plates of bainitic ferrite separated by thin layers of retained austenite. Compared to conventional austempering, the new method produced a much finer microstructure and a reduced amount of retained austenite. Moreover, the AT-2 variant further enhanced the homogeneity of the microstructure, promoting a higher fraction of thin ferrite plates. Mechanical tests revealed that the new heat treatment significantly improved the performance parameters of the material: hardness increased from 27 HRC to 32–35 HRC, tensile strength rose from 1027 MPa to 1220–1296 MPa, and yield strength from 683 MPa to 1033–1054 MPa. It was proved that, regardless of the initial microstructure of ADI, new processes exhibited comparable, enhanced mechanical properties, confirming their efficiency and universality.

## 1. Introduction

Austempered ductile iron (ADI) is a structural material widely used for machine parts and equipment in the agricultural, railway, automotive, and mining industries. Global cast iron production continues to grow steadily, at 20,000 tons per year on average. The microstructure of ADI consists of spheroidal graphite nodules embedded in a ferritic–austenitic matrix, known as ausferrite. This structure is obtained through a heat treatment sequence involving austenitisation followed by austempering. The ausferritic matrix provides ADI with relatively high mechanical properties, including tensile strengths of 850–1600 MPa, elongations exceeding 10%, as well as high impact strength and wear resistance [1,2,3,4,5,6,7,8]. The mechanical properties of ADI can be tailored through heat treatment, which affects the type, amount, and size of phases in the microstructure, including stable retained austenite that contributes to ductility [9,10,11,12,13,14,15,16]. Numerous advanced heat treatment methods have been developed for steels to refine microstructure and enhance the strength–ductility balance [17,18,19,20]. Some of these treatments have been adapted for ADI [21,22,23,24,25,26,27,28,29,30,31,32], such as multistep austempering [24,25,26,27,28], thermomechanical processing [22,29,30,31,32,33], and quenching and partitioning (Q&P) treatments [34,35,36]. Their common outcome is a highly refined, multiphase microstructure containing hard phases (martensite and/or bainitic ferrite) along with a significant proportion of stable retained austenite. Grain refinement increases strength [5,14,21,22,23] while control over the amount and stability of austenite improves ductility [9,14,32,33].

One important route to modify ADI microstructure while reducing processing time is staged austempering, including step-up (low–high) and step-down (high–low) methods [24,25,26,27,28]. In step-up austempering, the first stage is conducted at a lower temperature within the bainitic transformation range to generate a high density of ferrite nuclei. The second stage at a higher temperature promotes the growth of ausferrite, carbon diffusion, and austenite stabilization, producing a finer microstructure with higher stable austenite content, leading to increased strength (up to several hundred MPa) and fracture toughness compared with single-step processing at the same temperature [24,25,28]. In step-down processing, the initial high-temperature stage produces fewer ferrite nuclei, but their growth is more pronounced. The subsequent step at a lower temperature rises the degree of bainite transformation, leading to a reduction in the austenite content and an increase in its stability in the final microstructure. This method provides similar properties to single-step austempering carried out at a lower temperature, but with a shorter processing time [26,27].

Thermomechanical treatment, applied either during austenitisation [22,29] or austempering [30,31,32], increases defect density, which leads to the refinement of the microstructure and shortening the transformation time [29,30,31,32,33]. Plastic deformation during austempering (ausforming) additionally suppresses the martensitic transformation of unstable austenite during cooling, although deformation within the bainitic range can itself trigger martensite formation. These effects—microstructural refinement, increased dislocation density, and potential martensite presence—can substantially improve strength (even up to several hundred MPa), but ductility depends strongly on the chemical composition, deformation level, and martensite fraction [29,30,31,32].

Q&P processes are also attracting attention. In the quenching stage (Q), the material is cooled to a temperature T_Q_ between the martensite start (Ms) and finish (Mf) temperatures, producing partial martensitic transformation. In the partitioning stage (P), performed at a temperature T_P_ ≥ T_Q_ (up to ~450 °C), carbon diffuses from supersaturated martensite into austenite, stabilizing it without altering phase proportions. Appropriate Q&P parameters yield microstructures combining tempered martensite and carbon-enriched retained austenite, or mixtures of martensite, austenite, and ausferrite, providing high tensile strength and ductility. Inadequate partitioning (low temperatures or short durations) reduces stable austenite content, promoting unwanted martensitic transformation on cooling, which increases strength but lowers ductility [34,35]. Modified approaches replace quenching with short low-temperature austempering, followed by partitioning, producing nanometric to submicron ausferrite with high retained austenite content. Partitioning then continues bainitic transformation, refines ferrite plates, and reduces blocky austenite, enhancing the elongation from 3.6% to 5.2%, i.e., by about 50%, with only a slight reduction in strength [36]. Another development path, pursued in this study, is the application of nanobainitisation-based processes involving austempering at low temperatures near the Ms. Industrial practice distinguishes low austempering (250–300 °C for 1.5–4 h) and high austempering (350–400 °C for 0.5–2 h). Conventional ADI exhibits a feathery ferrite morphology with high austenite content and relatively coarse features. New approaches employ greater undercooling and optimized holding times to produce more refined microstructures. Nanobainitisation is performed slightly above Ms (Ms ~20 °C) for the full duration of bainitic transformation, which is longer than standard industrial practice. Lowering the temperature refines ferrite plates and austenite blocks, but also reduces austenite content [5,14,21]. This strengthens ADI but reduces ductility and toughness. Austenite formed at low temperatures may be mechanically less stable and may transform to martensite during deformation (TRIP effect), further increasing tensile strength [37]. However, insufficient carbon supersaturation can cause premature martensitic transformation, either during heat treatment or in service, severely reducing ductility. Ensuring proper carbon diffusion and austenite stabilization at lower temperatures requires extended austempering times, which may slightly influence ferrite and austenite morphology [14]. A critical holding time exists beyond which tensile strength is unaffected, although ductility and toughness may still vary. Low-temperature ausferrite also exhibits wear resistance comparable to martensitic hardened alloy steels [38], expanding its industrial applications.

Recent research has thus focused on modifying heat treatment to produce submicron or nanocrystalline ausferritic microstructures—fine bainitic ferrite plates separated by thin layers of stable retained austenite [10,14,16,23]—to improve strength and ductility. However, the influence of factors beyond austenitising and austempering conditions remains insufficiently studied. In the literature, there are no reports on the effect of preliminary isothermal quenching on ADI microstructure and properties. This work addresses that gap by examining pearlitic–ferritic and ausferritic initial states and their influence on the homogeneity, refinement, and mechanical properties (tensile strength, yield strength, impact toughness, and ductility) of nanocrystalline ausferritic ductile iron. The methodology also involved extended austempering times, sufficient to complete bainitic transformation without carbide precipitation, to achieve superior combinations of strength, toughness, and ductility in ADI—most studies had limited austempering times of up to 3–4 h (rarely exceeding 6 h) [39,40,41,42,43,44].

## 2. Materials and Methods

**Figure 1:**
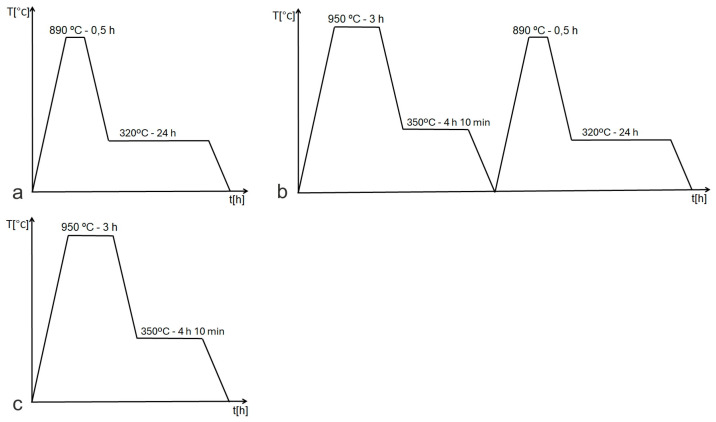
Heat treatment cycles for ADI: (**a**)—AT-1, (**b**)—AT-2, (**c**)—conventional.

Samples of commercial ductile iron were supplied by Polish Foundries JSC (Starachowice, Poland). The alloy was cast in Y-shaped ingots with a 25 mm base in accordance with the manufacturer’s specifications for heat-treated ductile iron castings. The chemical composition, determined using a Belec IN-SPECT spectrometer (Belec Spektrometrie Opto-Elektronik GmbH, Georgsmarienhütte, Germany), is presented in (Table 1). Various heat treatment variants were applied (Figure 1). In the first variant (AT-1), samples with an initial ferritic–pearlitic microstructure were austenitised at 890 °C for 0.5 h and then subjected to long-term austempering at 320 °C for 24 h. The austempering temperature lies within the lower bainitic transformation range and was selected based on dilatometric analysis to promote microstructural refinement; therefore, this process can be described as a nanostructuring treatment. At this temperature, the carbon diffusion rate is sufficient to enrich retained austenite, thereby increasing its stability.

The second variant (AT-2) consisted of two stages. First, a pre-heat treatment was carried out: samples were austenitised at 950 °C for 3 h, followed by isothermal holding at 350 °C for 4 h 10 min, producing a typical ausferritic microstructure. These samples were then re-austenitised at 890 °C for 0.5 h and subjected to long-term austempering at 320 °C for 25 h 43 min (nanostructurisation treatment). The purpose of this double treatment was to further refine the grain structure and improve mechanical properties. Prolonged and high-temperature austenitisation can increase the carbon concentration in the ADI matrix [45,46,47], resulting in higher retained austenite content and improved ductility. Higher carbon content can also extend the incubation time and reduce bainitic transformation kinetics [48], thereby broadening the process window [49]. Cyclic quenching is also known to refine grains and enhance mechanical properties [50,51,52,53].

All austenitisation treatments were conducted in a Czylok FCF-V70C/R gas-tight furnace (Czylok Ltd., Jastrzębie Zdrój, Poland). Cooling and isothermal holding were performed in a Czylok furnace equipped with a SiC fluidized bed agitated by air, with additional steam used during cooling.

**Figure 2:**
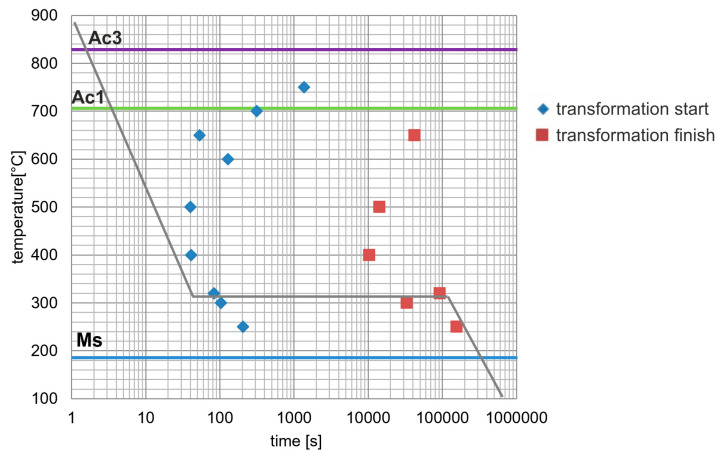
TTT diagram of examined ductile iron.

The heat treatment parameters were established using phase transformation data obtained from dilatometric tests carried out on a Bahr DIL 805 L dilatometer (Bähr-Thermoanalyse GmbH, Hüllhorst, Germany). Cylindrical specimens (10 mm length, 3 mm diameter) were heated in vacuum to 890 °C at 2 °C·s^−1^, held at temperature, and cooled at 50 °C·s^−1^ to the isothermal holding temperature (250–750 °C). Isothermal holding was conducted for 24–48 h, followed by helium gas cooling at 50 °C·s^−1^ to room temperature. Transformation start and finish times were determined from the dilatometric curves, and a time–temperature–transformation (TTT) diagram was constructed (Figure 2). The final isothermal dwell time corresponded to the completion of the ausferritic transformation.

For comparison, a conventional heat treatment, consisting of austenitisation at 950 °C for 3 h and isothermal holding at 350 °C for 4 h 10 min, was also performed.

Microstructural observations were carried out using a Hitachi S3500N scanning electron microscope (SEM) (Hitachi Ltd., Tokyo, Japan) and a JEOL JEM 1200 EX transmission electron microscope (TEM) (JEOL Ltd., Tokyo, Japan) in both bright-field (BF) and dark-field (DF) modes, combined with electron diffraction analysis. For SEM, specimens (10 × 10 × 3 mm) were ground with SiC paper up to 1200 grit, polished with 3 μm diamond suspension, and etched in 4% nital. SEM observations were performed in secondary electron mode. For TEM, disks 2.7 mm in diameter were sectioned, thinned to ~200 μm, ground to 100 μm, and ion-polished to perforation. Quantitative microstructural analysis (grain size, phase fraction) was performed using stereological methods. Ferrite plate and austenite layer thicknesses were calculated according to the stereological relation [54]:

(1) $$d=\frac{2}{\Pi }L$$

where *d* is the true plate thickness and *L* is the apparent width measured perpendicular to the intersection line of the interphase boundaries on the thin foil surface.

The amount and size of retained austenite blocks were estimated from SEM images using MicroMeter 1.0 software [55], following the procedure in Ref. [56]. The retained aus.tenite volume fraction was determined by X-ray diffraction (XRD) using a Rigaku SmartLab 3 kW diffractometer equipped with a 1D Dtex250 linear detector and a Cu X-ray tube, operating at 40 kV and 30 mA (Rigaku Corporation, Tokyo, Japan). Data were collected in the 2θ range of 20–100° with a step size of 0.02°. Prior to XRD, samples were repeatedly polished and etched in 4% nital to remove the deformed surface layer.

Mechanical properties were evaluated via Brinell hardness (HB), Rockwell hardness (HRC), impact toughness (KVC), and tensile testing. Hardness was measured on three samples (10 × 10 × 3 mm) for each treatment, with five indentations per sample. Tensile tests were performed in accordance with ISO 6892-1:2019 [57] on cylindrical specimens (6 mm diameter, gauge length L_0_ = 30 mm) using an MTS810 testing machine (MTS Systems Corporation, Eden Prairie, Minnesota, USA) equipped with a 100 kN load cell (subrange 0–50 kN) at a strain rate of 2.5 × 10^−4^ s^−1^. Three specimens per treatment were tested. Impact tests followed DIN EN 10045-1:1991-04 [58] using a Charpy Zwick/Roell RKP450 machine (ZwickRoell Group, Ulm, Germany) with 150 J capacity on 10 × 10 × 55 mm V-notched samples, with three specimens per treatment.

Fracture surfaces after tensile and impact tests were examined using the Hitachi S3500N SEM (Hitachi Ltd., Tokyo, Japan). The properties of ADI subjected to austempering with and without pre-heat treatment were compared with those of conventionally treated ductile iron (austenitisation at 950 °C for 3 h, isothermal holding at 350 °C for 4 h 10 min).

## 3. Results and Discussion

### 3.1. Dilatometric Analysis

**Figure 3:**
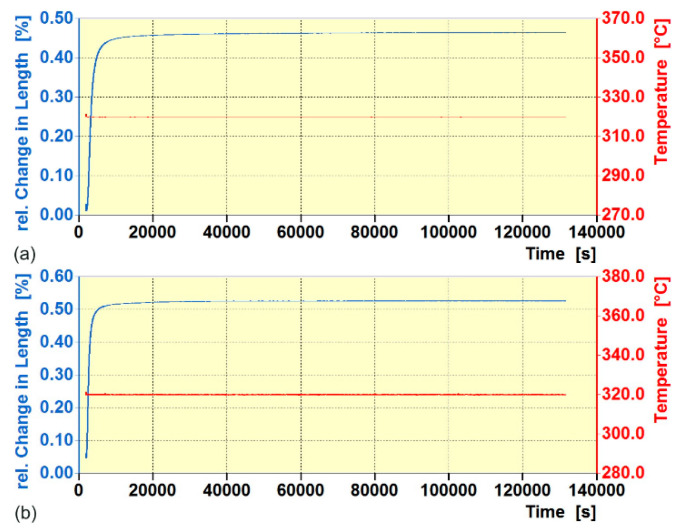
Dilatometric curves showing ΔL/L_0_ as a function of time for (**a**) AT-1 and (**b**) AT-2 samples during final austempering at 320 °C.

Figure 3 presents the dilatograms for single-step (AT-1, Figure 3a) and double-step (AT-2, Figure 3b) austempered ductile iron samples. The observed increase in sample length at a constant temperature can be attributed primarily to the bainitic phase transformation of supercooled austenite. This occurs because the alloy contains a high concentration of silicon, which suppresses cementite precipitation, as well as significant amounts of molybdenum, copper, and nickel, which collectively widen the so-called process window [59,60].

It is likely that the prolonged high-temperature austenitization used in these treatments increased the carbon content in the matrix. This, in turn, may have contributed to an extended incubation time, a reduced transformation rate [48], and an overall widening of the process window [49]. The dilatometric measurements showed that complete bainitic transformation was achieved after approximately 24 h for pearlitic–ferritic ductile iron and 25 h 43 min for ausferritic ductile iron. Extending the austempering time beyond these durations, even up to 36 h, did not cause further transformation of austenite into bainitic ferrite, indicating that the residual austenite remained thermally stable during prolonged exposure at the austempering temperature.

For conventional austempering of ductile iron, even at temperatures close to 300 °C, the processing time is typically limited to several hours. Analysis of dilatometric curves for some alloys has shown that such shorter durations can interrupt the austenite transformation at 50–60% completion. This leads to an incomplete ausferritic transformation and a microstructure with a substantial proportion of untransformed—and in some cases unstable—austenite. Due to the incomplete reaction phenomenon [61], even after nominally 100% transformation, a considerable amount of untransformed austenite may remain. While stable residual austenite enhances ductility, unstable austenite can transform into martensite during cooling or under applied stress, significantly reducing plasticity [12]. According to Putatunda and co-workers [12,62,63], a high fraction of stable residual austenite in ductile iron can be achieved even after very short austempering durations. However, other researchers have argued that during short austempering, the processing temperature has a stronger influence on the retained austenite content than the treatment time [64]. On the other hand, proper control of carbon concentration in austenite permits to obtain appropriate stability of austenite, which allows for the TRIP effect to occur, which increases the ductility of ADI. Alloying additions such as manganese and molybdenum promote segregation, which can locally reduce the carbon concentration and facilitate the shear transformation of austenite. Conversely, Mo, Ni, and Cu extend the duration of the first stage of bainitic transformation [60,65]. In the present study, austempering durations corresponding to the time required to reach the maximum transformation rate were deliberately chosen. This approach aimed to maximize the volume fraction of bainite and stable retained austenite, thereby suppressing martensitic transformation during cooling or straining, and improving hardness and mechanical properties.

### 3.2. Microstructural Characterization

**Figure 4:**
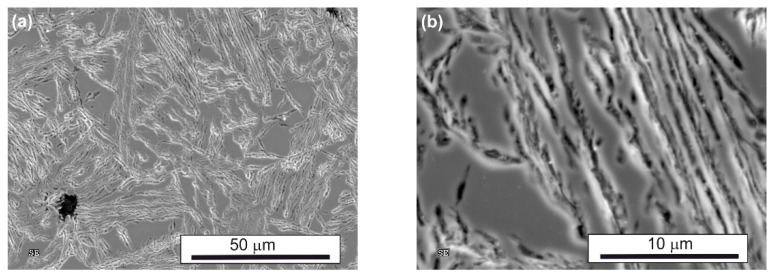
The microstructure of the ADI after conventional austempering: lower (**a**) and higher (**b**) magnifictaion.

SEM observations revealed a typical feathered ausferritic microstructure after conventional austempering, consisting of large blocks of retained austenite (up to 143 µm^2^—Table 2) and coarse, parallel ferrite plates (Table 3, Figure 4). In both AT-1 and AT-2 samples, austempered at lower temperatures, the microstructure was significantly finer than after conventional treatment. This refinement is attributed to greater supercooling of austenite, which increases the nucleation rate of ferrite and decreases the critical nucleus size and thus promotes grain refinement [21].

**Figure 5:**
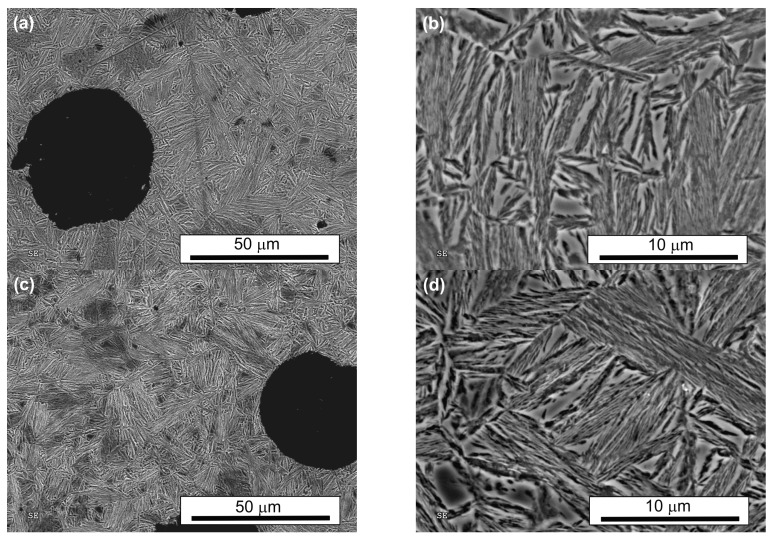
The microstructure of the ADI after single austempering (AT-1): the edge (**a**,**b**) and the core (**c**,**d**) of the sample.

**Figure 6:**
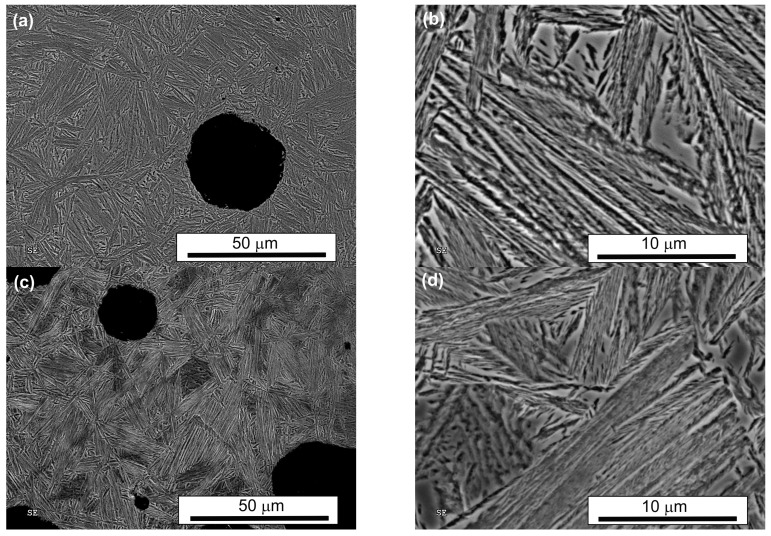
The microstructure of the ADI after double austempering (AT-2): the edge (**a**,**b**) and the core (**c**,**d**) of the sample.

During prolonged isothermal annealing, auto-catalytic nucleation of bainitic ferrite can also occur, further fragmenting the microstructure. In both AT-1 and AT-2 samples, the microstructure consisted of packets of thin, parallel ferrite plates separated by thin layers of retained austenite with no significant difference between the core and edge (Figure 5 and Figure 6). These packets and layers were oriented at various angles, with bainitic plates initiating at prior-austenite grain boundaries and growing into the austenite grains. Retained austenite was also present in blocky form, with a regular shape and feathered edges.

The total retained austenite content in both novel treatments (AT-1: ~21.7%; AT-2: ~22.4%, Table 4) was roughly half that of the conventional treatment (~41.7%). In AT-2 samples, ferrite plates were longer, while the number and average size of blocky retained austenite were slightly lower than in AT-1. The AT-2 microstructure was more homogeneous and less fragmented. It resembled the fine structure produced during low-temperature isothermal quenching [60,66].

**Figure 7:**
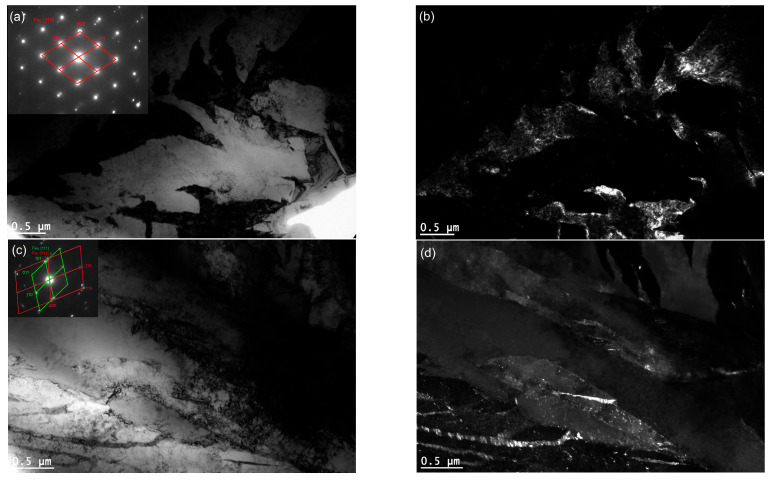
The microstructure of the pearlitic–ferritic ductile iron after conventional austempering: (**a**,**c**)—bright-field images, (**b**,**d**)—DF images from area (**a**,**c**) obtained with the use of austenite reflection.

**Figure 8:**
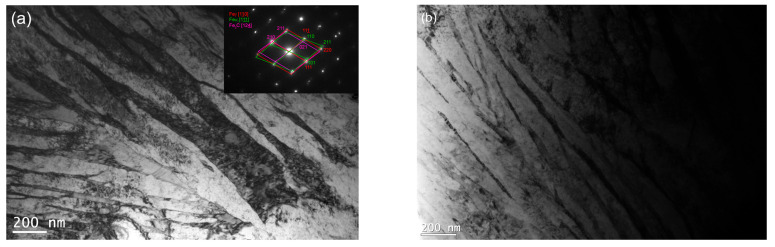
The microstructure of the pearlitic–ferritic ductile iron after single austempering (AT-1)—bright-field image (**a**,**b**).

**Figure 9:**
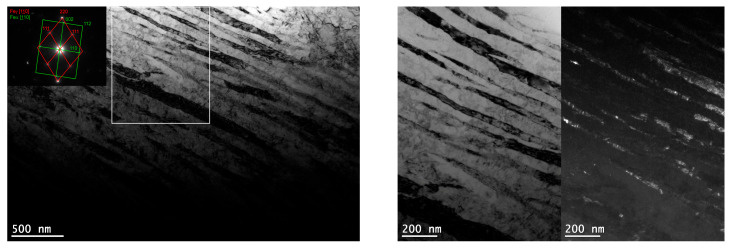
The microstructure of the ausferritic ductile iron after double austempering (AT-2)—DF image obtained with the use of austenite reflection.

**Figure 10:**
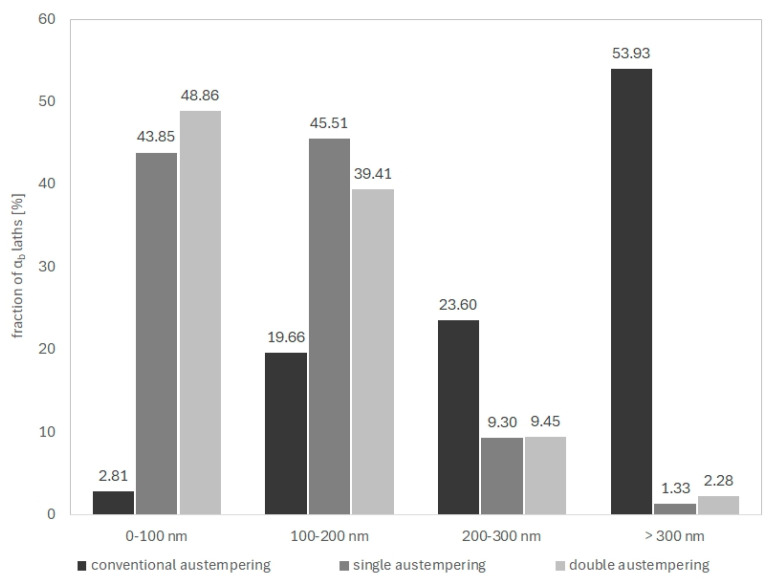
Distribution of size of ferrite plates in the microstructure of the ADI after conventional single (AT-1) and double (AT-2) austempering.

**Figure 11:**
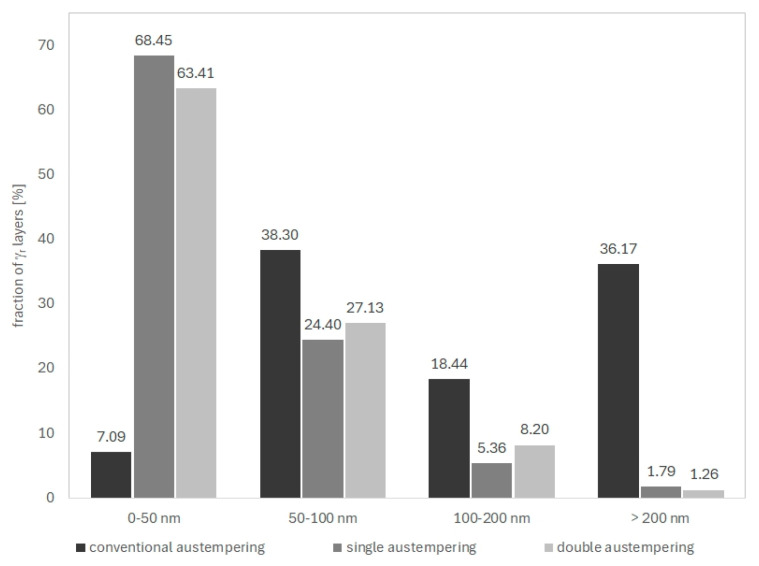
Distribution of size of residual austenite layers in the microstructure of the ADI after conventional single (AT-1) and double (AT-2) austempering.

TEM confirmed the feathered, coarse ausferritic structure after conventional austempering (Figure 7). The average ferrite plate thickness was 351 nm, and the average austenite layer thickness was 225 nm (Table 3); however, very large ferrite grains (>300 nm) and thick austenite layers (>200 nm) dominant. In contrast, both AT-1 and AT-2 microstructures consisted of nanoausferrite—bainitic ferrite plates with nanometric thickness, separated by very thin retained-austenite layers (Figure 8 and Figure 9). AT-2 samples had a greater fraction (~5%) of ferrite plates thinner than 100 nm (Figure 10) and fewer very thin (<50 nm) retained-austenite layers (Figure 11) compared to AT-1. Blocky austenite content in AT-2 was also about 5% lower. The greater homogeneity of the AT-2 microstructure is attributed to the pre-heat treatment, involving high-temperature, long-term austenitization before austempering—an innovative approach compared to cyclic martensitic quenching. Previous studies on steels and cast irons have shown that cyclic martensitic quenching refines austenite grains during subsequent austenitization, with the greatest effect after the first cycle [50,52,53]. To avoid the thermal stresses associated with martensite, this study replaces continuous quenching with austempering preceded by long-term, high-temperature austenitization—an entirely novel approach. High austenitization temperature yields large, homogeneous prior-austenite grains with increased carbon concentration. This results in reduced density of ferrite nucleation sites, long ferrite plates, as well as coarser austenite layers and blocks [14,67]. Moreover, the increased carbon content strengthens the austenite which in turn limits the thickening of ferrite plates during austempering. This can leave a significant fraction of untransformed austenite in the microstructure, increasing with higher austenitization temperature and time. The pre-heat treatment used in this work refined the initial microstructure and retained some residual austenite, facilitating easier nucleation during the second cycle and increasing carbon content and homogeneity. Consequently, AT-2 produced a more uniform microstructure with slightly thinner plates and slightly higher retained-austenite content than AT-1.

Cementite precipitates were observed in all heat-treated samples, forming as very thin layers at ferrite–austenite interfaces. Extending austempering time to 36 h did not affect their formation or growth [10,16] suggesting that cementite precipitation occurs well before bainitic transformation completion, regardless of Si, Cu, Mo, or Ni additions. Therefore, cementite presence is not necessarily linked to austenite decomposition.

### 3.3. Mechanical Properties and Fracture Surface Analysis

Hardness increased from 26.8 HRC in the conventional treatment to 37.2 HRC (AT-1, +38%) and 35.0 HRC (AT-2, +36%). Tensile strength rose from 1027 MPa to 1220 MPa (AT-1, +18%) and 1296 MPa (AT-2, +25%), while yield strength increased from 683 MPa to 1033 MPa (AT-1, +51%) and 1054 MPa (AT-2, +54%) (Table 5). However, ductility decreased: elongation dropped from 11% to 4.3% (AT-1, −61%) and 4.9% (AT-2, −55%), and impact strength fell from 13.3 J/cm^2^ to 9.0 J/cm^2^ (AT-1, −32%) and 8.3 J/cm^2^ (AT-2, −37%). The observed improvement in strength and the accompanying reduction in ductility after the AT-1 and AT-2 treatments can be attributed to microstructural changes caused by strong undercooling during austempering. The use of a low isothermal transformation temperature promotes intensive nucleation of bainitic ferrite while limiting its growth, leading to the formation of a nanoausferritic structure consisting of ferrite plates with an average thickness of approximately 120 nm and thin retained austenite films about 50 nm thick.

This refinement enhances the mechanical properties through grain boundary strengthening and the Hall–Petch effect, where grain boundaries act as barriers to the dislocation motion, causing dislocation pile-ups and consequently increased stress required for plastic deformation. Furthermore, the displacive nature of the bainitic transformation contributes to an increase in dislocation density within the matrix, additionally strengthening the material.

At the same time, the prolonged austempering time and low transformation temperature reduces the amount of retained austenite, particularly in the form of a large, blocky region, compared with the conventional treatment. Large austenite blocks are typically less carbon saturated and therefore more susceptible to deformation-induced transformation (TRIP effect). The suppression of this mechanism in smaller blocks and thin austenite films, together with the lower overall volume fraction of the ductile austenitic phase, leads to a decrease in elongation and impact toughness.

Moreover, high local stresses at ferrite–austenite interfaces and possible carbide precipitation along grain boundaries may act as crack initiation sites, promoting the formation of quasi-cleavage fracture surfaces. Consequently, while the nanostructured ausferritic matrix provides substantial strengthening, it simultaneously limits the material’s capacity for plastic deformation.

**Figure 12:**
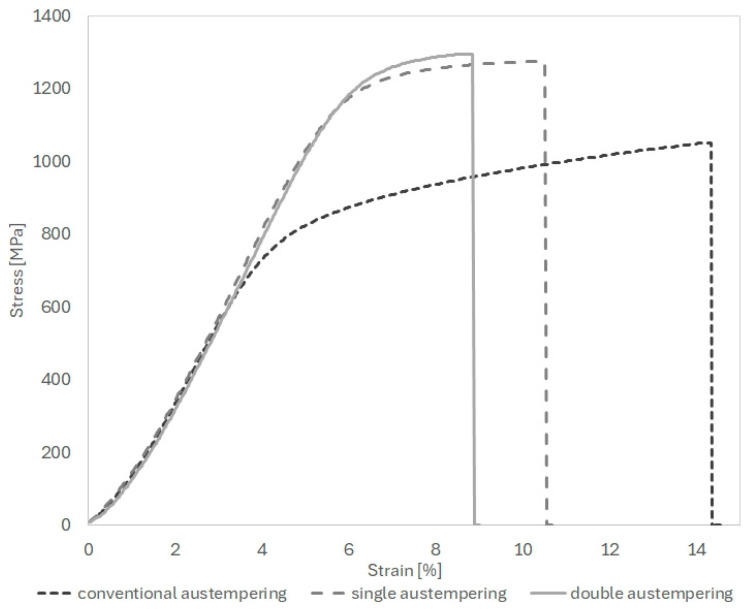
Representative stress–strain curve of the ductile iron after various heat treatments.

The pre-heat treatment prior to long-term austempering (double austempering, AT-2) did not significantly affect the mechanical parameters of the material (Table 5, Figure 12). The strength and ductility parameters of ductile iron after single (AT-1) and double (AT-2) austempering were similar, with slightly higher values for AT-2 and significantly higher than those obtained after conventional austempering. For the ADI AT-2 sample, the microstructural refinement is slightly greater, which is reflected in the slightly higher tensile strength and yield strength. In both samples, austenite content is very similar, indicating that the differences in mechanical behavior are mainly governed by the morphology. However, during tensile deformation, austenite may undergo strain-induced transformation (TRIP effect), locally increasing the strength of the matrix. Consequently, AT-2 combines improved microstructure and more effective strain hardening, leading to higher ultimate tensile strength despite its marginally lower hardness.

At the same time, a reduction in elongation and impact toughness was observed compared to conventional heat treatment. The tensile and yield strength values obtained after long-term austempering are considerably higher than those reported by other authors for materials with a similar chemical composition, subjected to one-step austempering at comparable temperatures [5,14]. However, extending the austempering time does not guarantee the same high tensile and yield strength achieved by other researchers through step-up processes (UTS around 1485 MPa, YS approximately 1245 MPa, with elongation of about 4.6%) [21,25,68].

**Figure 13:**
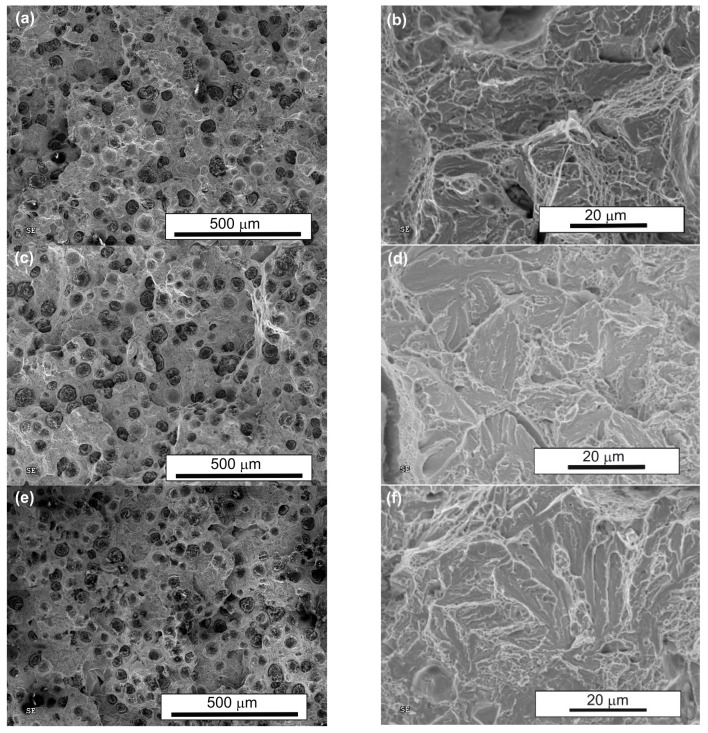
Fracture surfaces of heat-treated ADI after static tensile testing: (**a**,**b**)—conventional austempering; (**c**,**d**)—single austempering (AT-1); (**e**,**f**)—double austempering (AT-2).

**Figure 14:**
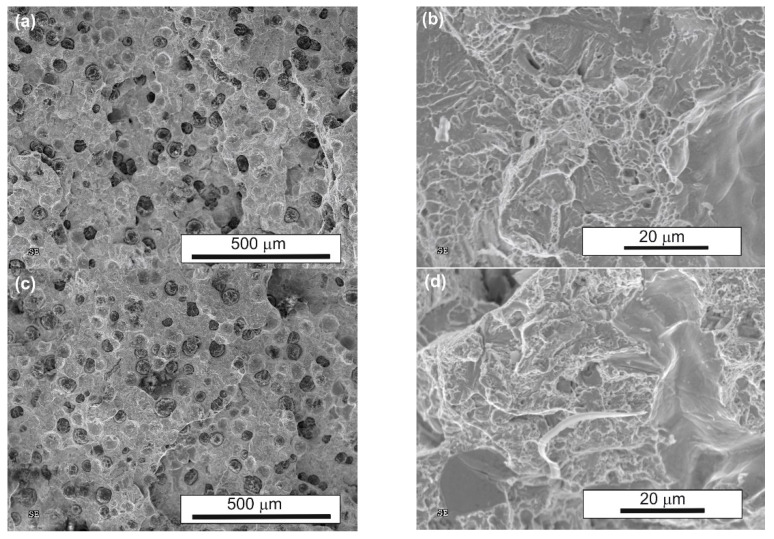

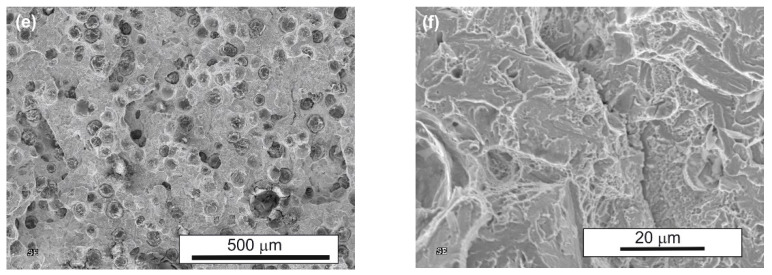
Fracture surfaces of heat-treated ADI after impact testing: (**a**,**b**)—conventional austempering; (**c**,**d**)—single austempering (AT-1); (**e**,**f**)—double austempering (AT-2).

To better understand the ductility and impact energy results, the fracture surfaces of the samples were examined after tensile tests (Figure 13) and after impact tests (Figure 14). All fracture surfaces of the tensile-tested samples exhibited a combination of ductile and quasi-cleavage characteristics, regardless of the heat treatment applied. The samples showed uniform deformation within the gauge length, without visible necking in the fracture region. SEM observations at low magnification revealed that the fracture surface of conventionally treated material was smoother compared to those of the samples after both single (AT-1) and double (AT-2) austempering. Differences became more evident at higher magnification: in all cases, dimples and quasi-cleavage facets were observed; however, fractures after conventional austempering exhibited a more pronounced ductile character, which was associated with a higher amount of retained austenite. Lowering the austempering temperature and extending the processing time resulted in microstructure refinement and a reduction in the amount of retained austenite, which enhanced strength, but also increased the number of quasi-cleavage facets, with the highest proportion observed after double (AT-2) austempering.

Similarly, SEM observations of fracture surfaces after impact tests revealed that all fractures displayed mixed ductile and quasi-cleavage features. The morphology of the fracture surfaces was very similar to that observed after the tensile tests. Some secondary cracks were noted on the fractures of the samples after both single (AT-1) and double (AT-2) austempering.

## 4. Conclusions

This work investigated the effects of new heat treatments involving long-term austempering at reduced temperatures on the microstructure and properties of ADI. The influence of the initial microstructure prior to austenitization, as well as the austenitization parameters on phase transformations during austempering and the final microstructure and mechanical properties of ADI, was analyzed. The obtained results were compared with those obtained for ADI after conventional austempering. The relationships between the heat treatment parameters, the initial microstructure, and the resulting microstructure and mechanical properties of austempered ductile iron provide a basis for optimizing ADI heat treatment strategies. This issue has not been sufficiently studied and described in the literature to date. The main conclusions are as follows:

1. Long-term austempering carried out at temperatures within the lower range of bainitic transformation resulted in pronounced microstructural refinement compared with conventional austempering. The applied process can be classified as a nanostructurisation heat treatment for ADI.
2. Double-step austempering (AT-2) resulted in a finer and more homogeneous microstructure, characterized by extremely thin ferrite plates (<100 nm) and a reduced fraction of blocky austenite compared with the single-step treatment (AT-1).
3. Both long-term austempering treatments reduced the retained austenite content from approximately 42% to about 22%, increasing hardness from 27 HRC to 32–35 HRC, tensile strength from 1027 MPa to 1220–1296 MPa, and yield strength from 683 MPa to 1033–1054 MPa, while reducing total elongation from 11% to 4.3–4.9% and V-notched impact toughness from 13 J/cm^2^ to 8–9 J/cm^2^.
4. Overall, both AT-1 and AT-2 treatments demonstrated clear advantages over conventional austempering by producing a finer and mechanically stronger nanoausferritic matrix.
5. Long-term austempering is suitable for ADI components requiring high strength and low material costs, provided that they are not subjected to significant dynamic loading. This work demonstrates the effectiveness of controlling the initial microstructure and applying long-term, low-temperature austempering to enhance the mechanical properties of ADI, representing a novel pathway for optimizing its heat treatment.

## Figures and Tables

**Table 1:** Chemical composition of investigated ductile iron.

Element	C	Si	Mn	Mg	Ni	Mo	Cu	Cr	P	S	Fe
wt. %	3.5	2.54	0.16	0.047	1.4	0.24	0.5	0.03	0.04	0.013	balance

**Table 2:** Area of blocks of residual austenite in the microstructure of ductile iron after various austempering—micrometer estimation.

	ADI After Conventional Austempering	ADI After Single Austempering (AT-1)		ADI After Double Austempering (AT-2)	
	average	core	edge	core	edge
Minimum [µm^2^]	1	0.02	0.44	0.4	0.74
Maximum [µm^2^]	143	22	43.47	13	24.54
Average [µm^2^]	14 ± 5	3.9 ± 0.9	3.81 ± 0.96	3.1 ± 0.9	4.16 ± 0.98

**Table 3:** Results of the stereological measurements of the thickness of austenite layers and bainitic ferrite plates in the ADI after various heat treatments.

Thickness of Structural Element	ADI After Conventional Austempering		ADI After Single Austempering (AT-1)		ADI After Double Austempering (AT-2)	
	Ferrite	Austenite	Ferrite	Austenite	Ferrite	Austenite
Minimum [nm]	70	30	24	9	15	8
Maximum [nm]	977	1192	686	420	434	268
Average [nm]	351 ± 28	225 ± 13	124 ± 8	47 ± 5	117 ± 8	52 ± 4

**Table 4:** The amount of residual austenite in the matrix of ductile iron after various austempering—micrometer estimation, XRD measurements.

	ADI After Conventional Austempering	ADI After Single Austempering (AT-1)	ADI After Double Austempering (AT-2)
The amount of blocky residual austenite [%] (micrometer)	28.9 ± 5.5	17.4 ± 3.3	12.8 ± 2.3
Total amount of residual austenite (XRD) [%]	41.7	21.7	22.4

**Table 5:** The mechanical properties of the ductile iron after various heat treatments.

	ADI After Conventional Austempering	ADI After Single Austempering (AT-1)	ADI After Double Austempering (AT-2)
HRC	27 ± 4	37 ± 4	35 ± 5
HB	266 ± 12	349 ± 38	331 ± 45
R_0.2_ [MPa]	683 ± 7	1033 ± 28	1054 ± 22
R_m_ [MPa]	1027 ± 28	1220 ± 30	1296 ± 2
A_5_ [%]	11 ± 0.3	4.3 ± 2.9	4.9 ± 0.2
KVC [J/cm^2^]	13.3 ± 1.2	9 ± 0	8.3 ± 1.2

HRC—Rockwell hardness, HB—Brinell hardness, R_0.2_ [MPa]—yield strength, R_m_ [MPa]—tensile strength, A_5_ [%]—total elongation, KVC—V-notch impact toughness.

## Data Availability

The raw data supporting the conclusions of this article will be made available by the authors on request.

## Abbreviations

The following abbreviations are used in this manuscript:

ADI	Austempered ductile iron
Q&P	Quenching and partitioning
Ms	Martensitic start temperature
Mf	Martensitic finish temperature
T_Q_	Quenching temperature
T_P_	Partitioning temperature
AT-1	Single austempering
AT-2	Double austempering
TTT	Time-temperature-transformation
Ac1	Temperature at which austenitic transformiation starts (during heating)
Ac3	Temperature at which austenitic transformiation ends (during heating)
SEM	Scanning electron microscope
TEM	Transmission electron microscope
BF	Bright-field observation
DF	Dark-field observation
SE	Secondary electron
XRD	X-ray diffraction
d	Thickness of a plate
L	Width of a plate
HB	Brinell hardness
HRC	Rockwell hardness
KVC	impact toughness
∆L/L_0_	Change in dilatometric sample length
R_0.2_	Yield strength
R_m_	Tensile strength
A_5_	Total elongation
